# Brazilian Portuguese translation and cross-cultural adaptation of the Withdrawal Assessment Tool-Alpha 2 Agonist

**DOI:** 10.62675/2965-2774.20260065

**Published:** 2026-07-24

**Authors:** José Colleti, Roberta Esteves Vieira de Castro

**Affiliations:** 1 Hospital Israelita Albert Einstein Department of Pediatrics São Paulo SP Brazil Department of Pediatrics, Hospital Israelita Albert Einstein - São Paulo (SP), Brazil.; 2 Universidade do Estado do Rio de Janeiro Hospital Universitário Pedro Ernesto Department of Pediatrics Rio de Janeiro RJ Brazil Department of Pediatrics, Hospital Universitário Pedro Ernesto, Universidade do Estado do Rio de Janeiro - Rio de Janeiro (RJ), Brazil.

According to the 2022 Society of Critical Care Medicine's PANDEM guidelines, dexmedetomidine is favored as a primary sedative for critically ill pediatric patients who are mechanically ventilated or recovering from cardiac surgery with an expectation of swift extubation.^(1)^ However, despite its widespread clinical adoption, standardized tools to track iatrogenic alpha-2 agonist (A2A) withdrawal are currently lacking. When patients experience dexmedetomidine withdrawal, documented symptoms typically include agitation, elevated blood pressure, accelerated heart rate, and, on rare occasions, seizures.^(2)^

To address this gap, Solodiuk et al. developed and preliminarily tested the Withdrawal Assessment Tool-Alpha 2 Agonist (WAT-A2A), a validated instrument to monitor withdrawal symptoms from dexmedetomidine and clonidine in acutely ill children.^(3)^ The WAT-A2A provides clinicians with an objective measure to quantify withdrawal symptoms, facilitate A2A weaning, and reduce unnecessary variability in clinical practice.^(3)^

## METHODS

Recognizing the importance of this tool for Brazilian pediatric intensive care units (ICUs), the Brazilian Research Network in Pediatric Intensive Care (BRnet-PIC) collaborated with the authors of the original instrument to develop a culturally adapted Brazilian Portuguese version (BP-WAT-A2A). This translation adhered to established guidelines for international translation and cross-cultural adaptation.^(4)^ The adaptation process was particularly critical given the linguistic and cultural nuances of Brazilian Portuguese, which differ from those of other Portuguese-speaking countries.

The translation process involved two independent Brazilian pediatric intensive care physicians, each with over 10 years of clinical experience and fluency in English. They produced two initial translations of the tool and its instructions, which were then reconciled through a consensus process to create "version 1." To ensure conceptual equivalence, "version 1" was back-translated into English by an independent, highly qualified translator. The back-translated version was reviewed and approved by the original WAT-A2A creators, confirming the preservation of the tool's clinical intent and accuracy.

A limitation of this study is that the translated tool was not pilot-tested among Brazilian pediatric intensivists. We skipped that step because we believe the tool is very clear and straightforward.

## RESULTS

This study successfully delivers the cross-culturally adapted Brazilian Portuguese version of the WAT-A2A (Table 1). Intensive care physicians across the country are encouraged to integrate this tool into their routine clinical practice.

**Table 1:** Brazilian Portuguese version of the Withdrawal Assessment Tool-Alpha 2 Agonist

**Data**															
**Hora**															
**Informações do prontuário do paciente, antes do início do desmame do A2A**															
FC basal: +20%: +50%:															
PA diastólica basal: +20%: +50%:															
**Observação por 2 minutos antes do estímulo**															
**FC atual**															
**FC comparada à basal**															
	Igual ou próxima à basal = 0														
	≥ 20% = 1														
	≥ 50% = 2														
**PA diastólica atual**															
**PA diastólica comparada à basal**															
	Igual ou próxima à basal = 0														
	≥ 20% = 1														
	≥ 50% = 2														
**Comportamento**															
	Dormindo/acordado/calmo ou SBS = 0 = 0														
	Acordado/agitado ou SBS ≥ +1 = 1														
**Tremor**															
	Nenhum/leve = 0														
	Moderado/intenso = 1														
**Pontuação total (0 - 6)**															

**Instruções da Ferramenta de Avaliação de Abstinência – Alfa 2 Agonist (WAT-A2A)**
– Inicie a pontuação da WAT-A2A a partir do primeiro dia do desmame em pacientes que receberam doses de dexmedetomidina e/ou clonidina, incluindo adesivo transdérmico por > 5 dias. A WAT-A2A deve ser realizada pelo uma vez a cada 12 horas (ex: às 8h e às 20h +/- 2 horas). Continue a pontuar duas vezes por dia até 24 horas após a última dose de dexmedetomidina ou 72 horas após a última dose de clonidina.
**Antes de iniciar o desmame do A2A, documente a FC e PAD basais do paciente utilizando os dados dos últimos 5 dias do prontuário.**
– Este é o valor mais frequente de FC e PAD. Elimine períodos de dor, agitação ou eventos que alteram os sinais vitais. Observe o intervalo de valores +20% e +50% (valor basal x 1,2 = +20%; valor basal x 1,5 = +50%)
**Antes de estimular o paciente, observe-o por 2 minutos e registre o seguinte:**
– **FC atual:** pontue zero se a FC for igual ou próxima à basal. Pontue 1 se a FC aumentou ≥20%, mas menos que 50%. Pontue 2 se a FC aumentou ≥ 50%. – **PAD atual:** pontue zero se a PAD for igual ou próxima à basal. Pontue 1 se a PAD aumentou ≥20%, mas menos que 50%. Pontue 2 se a PAD aumentou ≥ 50%. – **Comportamento:** pontue zero se o paciente estiver dormindo, acordado ou calmo ou SBS = 0. Pontue 1 se o paciente estiver acordado e agitado ou SBS ≥ 1. – **Tremor:** pontue zero se o paciente não apresentou tremores ou apresentou apenas tremores ocasionais. Pontue 1 se o paciente apresentou tremores persistentes.
**Some os quatro itens para a pontuação total WAT-A2A (0-6).** Observe as tendências numéricas ao longo do tempo. FC - frequência cardíaca; PA - pressão arterial; SBS - State Behavioral Scale; PAD - pressão arterial diastólica. Source: Translated from: Solodiuk JC, Donado C, Wickerham L, Goodyear L, Hayes J, Mortell RE, et al. Development and preliminary testing of the withdrawal assessment tool-alpha 2 agonist: an assessment instrument for monitoring iatrogenic withdrawal symptoms in children receiving an alpha-2 agonist. Pediatr Crit Care Med. 2025;26(1):e67-76. © 2023 Jean C. Solodiuk and Martha A.Q. Curley Translated from English to Portuguese by Roberta Esteves Vieira de Castro and José Colleti Jr. (February 2025) Made available under a Creative Commons Attribution - Non Commercial - No Derivatives 4.0 International Deed: https://creativecommons.org/licenses/by-nc-nd/4.0/

## COMMENTS

To be effective in a clinical setting, evaluation tools must demonstrate validity, reliability, ease of administration, and distinct clinical utility, while also adapting to patient developmental stages and individual baseline variations. Employing a validated metrics system – such as the WAT-A2A – to measure A2A withdrawal signs helps streamline medication weaning and minimizes inconsistent care patterns. Consequently, this translated instrument aims to standardize the tracking of A2A withdrawal among critically ill pediatric patients, ultimately elevating healthcare standards and patient outcomes within Brazilian intensive care units.
